# Role of movement in long-term basal ganglia changes: implications for abnormal motor responses

**DOI:** 10.3389/fncom.2013.00142

**Published:** 2013-10-23

**Authors:** Nicola Simola, Micaela Morelli, Giuseppe Frazzitta, Lucia Frau

**Affiliations:** ^1^Section of Neuropsychopharmacology, Department of Biomedical Sciences, University of CagliariCagliari, Italy; ^2^Center of Excellence for Neurobiology of Dependence, University of CagliariCagliari, Italy; ^3^National Council of Research (CNR), Institute of NeuroscienceCagliari, Italy; ^4^Department of Parkinson Disease Rehabilitation, “Moriggia-Pelascini” Hospital, Gravedona ed Uniti (Como)Italy; ^5^Fondazione Europea Ricerca Biomedica (FERB), “S.Isidoro” Hospital, Trescore BalnearioItaly

**Keywords:** priming, movement, immobilization, *zif-268*, dynorphin, 6-OHDA, striatonigral, Parkinson’s disease

## Abstract

Abnormal involuntary movements (AIMs) and dyskinesias elicited by drugs that stimulate dopamine receptors in the basal ganglia are a major issue in the management of Parkinson’s disease (PD). Preclinical studies in dopamine-denervated animals have contributed to the modeling of these abnormal movements, but the precise neurochemical and functional mechanisms underlying these untoward effects are still elusive. It has recently been suggested that the performance of movement may itself promote the later emergence of drug-induced motor complications, by favoring the generation of aberrant motor memories in the dopamine-denervated basal ganglia. Our recent results from hemiparkinsonian rats subjected to the priming model of dopaminergic stimulation are in agreement with this. These results demonstrate that early performance of movement is crucial for the manifestation of sensitized rotational behavior, indicative of an abnormal motor response, and neurochemical modifications in selected striatal neurons following a dopaminergic challenge. Building on this evidence, this paper discusses the possible role of movement performance in drug-induced motor complications, with a look at the implications for PD management.

Motor complications induced by dopamine replacement therapy (DRT) are the major untoward effects associated with the pharmacologic management of Parkinson’s disease (PD). These complications include end-of-dose deterioration, motor fluctuations, and abnormal motor responses, the latter being very disabling and severely limiting the patient’s quality of life. Results obtained in experimental animal models of PD have indicated that pulsatile stimulation of dopamine receptors following DRT is a key step in the emergence of abnormal motor responses, and that these untoward effects are associated with a malfunction in the signal transduction pathway of the dopamine D_1_ receptor (Gerfen et al., 1990; Nutt, 2007; Santini et al., 2008; Guigoni and Bezard, 2009; Stocchi, 2009). Nevertheless, the precise mechanisms that underlie abnormal motor responses caused by DRT are still to be elucidated.

Recent findings have suggested that the generation of aberrant procedural memories in striatal motor circuits could participate in the manifestation of abnormal motor responses associated with DRT (Calon et al., 2000; Pisani et al., 2005; Jenner, 2008; Simola et al., 2009; Frau et al., 2013). Thus, the striatum plays a major role in processes such as integration of motor signals, acquisition of motor habits, and execution of motor programs, which are all critically regulated by dopamine (Mink, 1996; Packard and Knowlton, 2002; Gerdeman et al., 2003; Tang et al., 2007; Willuhn and Steiner, 2008). Starting from these premises, it has been hypothesized that the dopamine-denervated striatum fails to properly process motor information, and that this may result in an overload of striatal motor circuits following the performance of movement stimulated by drugs that activate dopamine receptors (Picconi et al., 2005; Pisani et al., 2005). This process, in turn, would promote pathologic motor learning, and eventually the onset of abnormal motor responses, such as dyskinesia (Picconi et al., 2005; Jenner, 2008). Therefore, the performance of movement might itself play a role in the emergence of abnormal motor responses caused by DRT. Interestingly, studies in both dopamine-denervated experimental animals and PD patients provide support to this view, by showing that physical activity may influence the severity of abnormal motor responses triggered by repeated administration of dopaminergic drugs (Reuter et al., 1999, 2000; Frazzitta et al., 2012; Aguiar et al., 2013). Moreover, recent evidence obtained in an experimental model of abnormal motor responses in hemiparkinsonian rats has provided a direct demonstration of an important role of movement performance in the emergence of these untoward effects (Simola et al., 2009; Frau et al., 2013).

## Critical evaluation of abnormal motor responses in experimental animal models of PD

Studies in experimental animals have dramatically contributed to the modeling of abnormal motor responses induced by dopaminergic drugs and elucidation of their mechanisms, and important results in this sense have been obtained in the unilaterally 6-hydroxydopamine (6-OHDA)-lesioned rat. Briefly, this animal model is characterized by a hemiparkinsonism subsequent to the infusion of 6-OHDA in the nigrostriatal pathway, which manifests as unilateral forelimb akinesia (Simola et al., 2007). Moreover, when treated with drugs that stimulate dopamine receptors, 6-OHDA-lesioned rats display a characteristic contralateral rotational behavior directed away from the site of toxin infusion, which is indicative of the antiparkinsonian effectiveness of the drug (Deumens et al., 2002; Simola et al., 2007). However, it is worth emphasizing that the repeated administration of drugs that stimulate dopamine receptors induces a sensitization in contralateral rotational behavior which reproduces the same biochemical changes observed in rats displaying dyskinetic-like abnormal involuntary movements (AIMs). In fact, abnormal motor responses induced by repeated treatment with dopaminergic drugs can be modeled in 6-OHDA-lesioned rats by measuring two types of behaviors: AIMs and sensitization in contralateral rotational behavior. AIMs consist of repetitive and purposeless movements of limbs and trunk, and are a reliable rodent model of human dyskinesias (Cenci et al., 1998; Lindgren et al., 2007). Sensitization in contralateral rotational behavior is also indicative of abnormal motor responses to dopaminergic drugs, since the intensity of this phenomenon directly correlates with the pro-dyskinetic potential of these drugs (Henry et al., 1998; Pinna et al., 2006; Carta et al., 2008).

With regard to sensitization in contralateral rotational behavior, it is worth mentioning the priming model. Priming involves a first administration of a dopamine D_1_/D_2_ receptor agonist (induction phase) that stimulates contralateral rotational behavior, followed, 3 days later, by the administration of a highly dyskinetic D_1_ receptor agonist (expression phase), at otherwise scarcely effective doses on rotational behavior (Morelli et al., 1989). Primed hemiparkinsonian rats display a vigorous contralateral rotational behavior on the expression phase, which is associated with neurochemical modifications in the striatum that are peculiar to experimental paradigms of prolonged administration of dopaminergic drugs (Pollack et al., 1997; van de Witte et al., 1998; Simola et al., 2007; Scholz et al., 2008; Nadjar et al., 2009). This evidence, therefore, indicates that the priming model is highly valuable for investigating the mechanisms that underlie abnormal motor responses to dopaminergic drugs in hemiparkinsonian rats.

## Movement performance following initial dopaminergic stimulation enables the manifestation of sensitized rotational behavior in primed hemiparkinsonian rats

**Figure 1:**
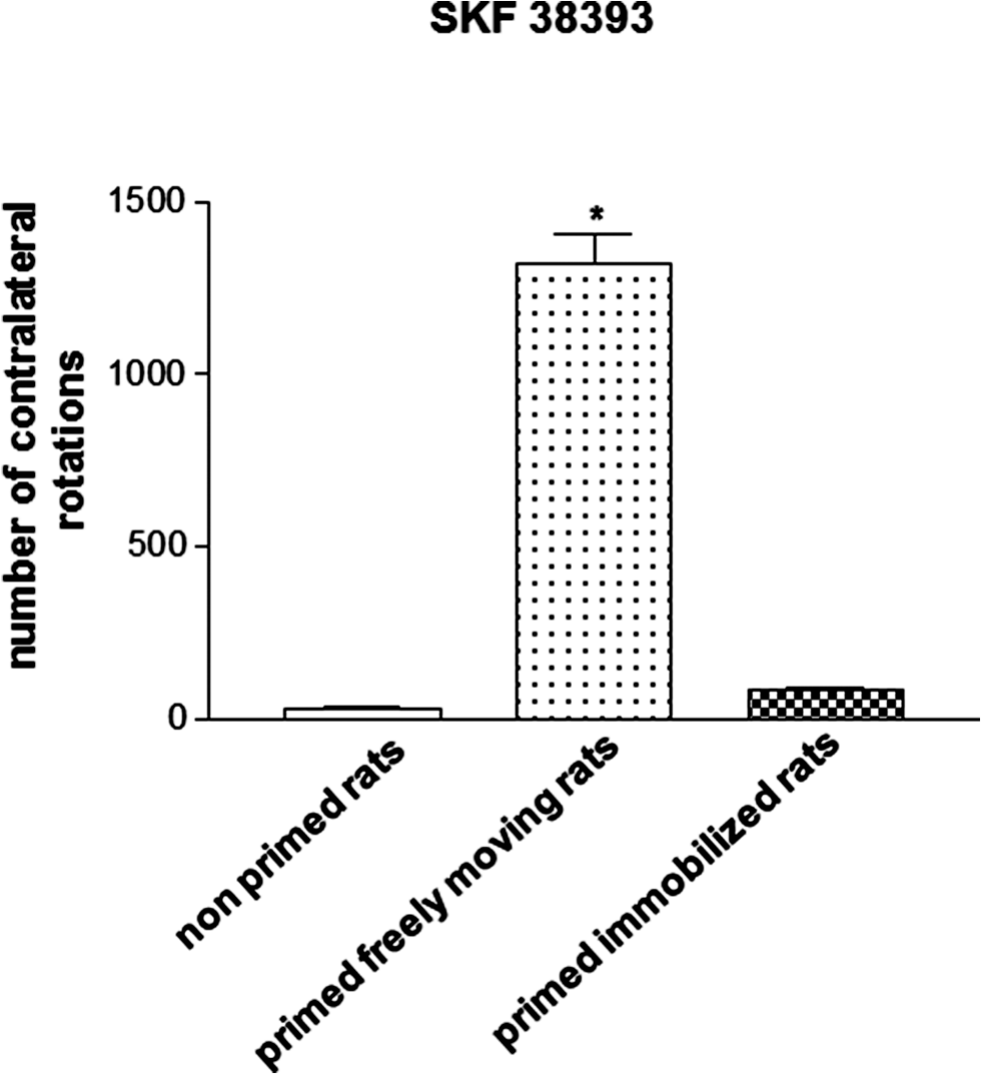
**Magnitude of contralateral rotational behavior stimulated by acute SKF 38393 (3 mg/kg s.c.) in primed rats, recorded for 2 h**. Priming induction was performed with apomorphine (0.2 mg/kg s.c.), and was followed by priming expression with SKF 38393 (3 mg/kg s.c.), 3 days later. Rats were either allowed to rotate or were immobilized during priming induction. *p < 0.05 vs. non-primed rats and primed immobilized rats.

Hemiparkinsonian 6-OHDA-lesioned drug-naïve rats that are treated with the D_1_/D_2_ agonist apomorphine (0.2 mg/kg, s.c.) during priming induction and left to rotate freely in response to the drug exhibit a marked contralateral rotational behavior when challenged 3 days later with the D_1_ receptor agonist 1-Phenyl-2,3,4,5-tetrahydro-(1H)-3-benzazepine-7,8-diol (SKF 38393, 3 mg/kg, s.c.), which is indicative of an abnormal motor response (Morelli et al., 1989; Simola et al., 2009). This behavioral effect of SKF 38393 has been found to be almost completely abolished in rats subjected to the same pharmacologic treatment that were immobilized for 1 h in a restrainer apparatus during priming induction, so that they could not perform rotational behavior in response to apomorphine (Figure 1; Simola et al., 2009). Importantly, immobilization has been demonstrated to suppress priming expression only when imposed concomitantly to apomorphine administration, but not immediately before or immediately after priming induction, therefore excluding a non-specific effect of immobilization (Simola et al., 2009). Moreover, the influence of immobilization on the effects of SKF 38393 has been shown not to be affected by the elevation in stress hormones that is associated with this procedure. This is clearly demonstrated by the finding that immobilization during priming induction retained the suppressant effects on priming expression even in rats treated with metyrapone (Simola et al., 2009), which prevents the elevation in stress glucocorticoid hormones that may be caused by immobilization (Calvo and Volosin, 2001). This finding is very important, since previous evidence has demonstrated that stress may have a profound influence on the behavioral and neurochemical effects of movement performance (Howells et al., 2005). Taken together, these findings indicate that the suppression of priming expression in rats immobilized during priming induction is attributable solely to the fact that immobilization prevented rats from performing movement in response to the initial dopaminergic stimulation (Simola et al., 2009). Therefore, the results obtained in hemiparkinsonian rats subjected to the priming model have provided the first direct demonstration that the performance of drug-stimulated movement may be crucial for the later emergence of abnormal motor responses to repetitive administration of dopaminergic drugs.

## Motor performance following initial dopaminergic stimulation triggers neurochemical modifications in selected striatal efferent neurons of primed hemiparkinsonian rats

In order to evaluate whether or not movement performance triggers changes indicative of neuronal long-term modifications, several biochemical and molecular parameters have been evaluated in the striatum of hemiparkinsonian drug-naïve and primed rats. The results obtained have demonstrated that both sensitized rotational behavior on the expression of priming and dyskinetic-like movements in dopamine-denervated animals repeatedly treated with dopaminergic drugs are associated with long-term changes in the production of striatal cyclic adenosine monophosphate (cAMP; Pinna et al., 1997), phosphorylation of dopamine- and cAMP-regulated neuronal phosphoprotein (DARPP-32; Santini et al., 2007), and expression of mRNAs encoding for different proteins and immediate early genes (IEGs; Barone et al., 1994; Cenci et al., 1998, 2009; Crocker et al., 1998; van de Witte et al., 1998; Carta et al., 2003, 2008; Aubert et al., 2005). In this regard, the IEG *zif-268* has recently been shown to be a useful marker of neuronal modification that involves striatal efferent pathways in animal models of abnormal motor responses (Carta et al., 2008, 2010).

*zif-268* (also known as *Egr-1*, *Krox-24*, *NGFI-*A, or *Zenk*) belongs to a class of inducible IEGs that encode regulatory transcription factors, and that have been implicated in diverse processes in a variety of cell types, including cell growth, differentiation, and apoptosis in response to extracellular stimuli (Gashler and Sukhatme, 1995). In the brain, *zif-268* mRNA and protein are constitutively expressed in several areas, such as the neocortex and hippocampus, and, of great importance in PD, the striatum (Christy et al., 1988; Mack et al., 1990; Schlingensiepen et al., 1991; Worley et al., 1991). Moreover, *zif-268* can be rapidly and transiently induced by a variety of pharmacologic and physiologic stimuli, including neurotransmitters, growth factors, peptides, depolarization, seizures, ischemia, and brain injury or cellular stress (Gashler and Sukhatme, 1995; Beckmann and Wilce, 1997). As mentioned above, *zif-268* may represent a useful and sensitive neurochemical marker in the evaluation of abnormal motor and neuronal responses associated with the onset of dyskinesia in dopamine-denervated animals treated with dopaminergic drugs (Carta et al., 2005, 2008). Thus, drugs such as L-3,4-dihydroxyphenylalanine (L-DOPA) and SKF 38393, which induce severe dyskinetic-like movements, markedly increase the levels of *zif-268* mRNA in striatal neurons, after both acute and subchronic treatment (Carta et al., 2005, 2008, 2010). In contrast, treatment with drugs that elicit a scarce dyskinetic-like response, such as ropinirole, do not produce any elevation in *zif-268* (Carta et al., 2010). Notably, the expression of mRNA encoding for *zif-268* was selectively increased in the direct striatonigral pathway, which seems to be the pathway most involved in development of dyskinetic movements (Carta et al., 2010).

**Figure 2:**
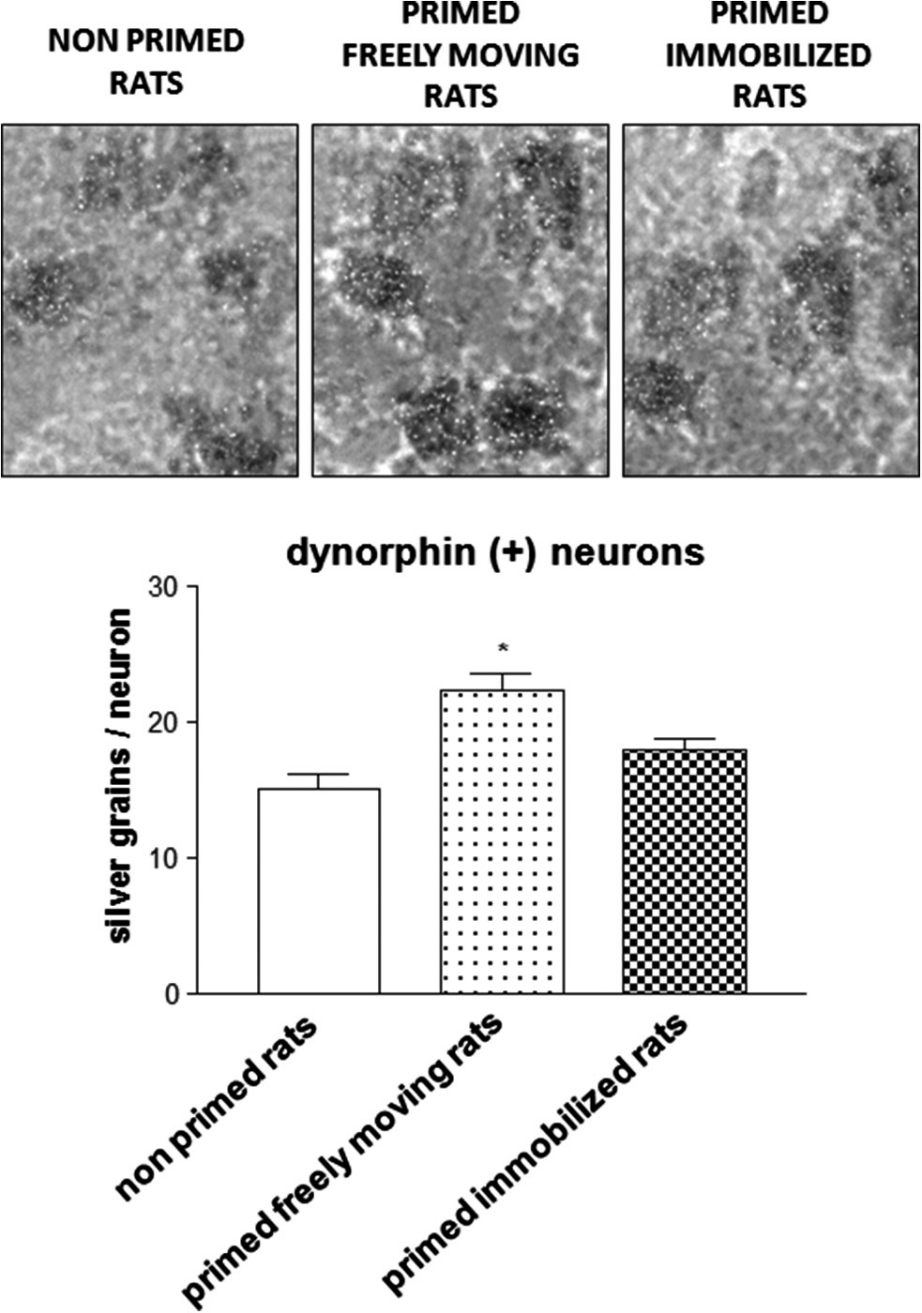
**Photomicrograph and histogram showing ***zif-268*** mRNA expression as number of silver grains/neuron in dynorphin(+) striatonigral neurons from the 6-OHDA-lesioned dorsolateral striatum of rats subjected to priming**. Priming induction was performed with apomorphine (0.2 mg/kg s.c.), and was followed by priming expression with SKF 38393 (3 mg/kg s.c.), 3 days later. Rats were either allowed to rotate or were immobilized during priming induction. Rats were sacrificed 2 h after SKF 38393 administration to perform *in situ* hybridization studies. *p < 0.05 vs. non-primed rats and primed immobilized rats.

Similar to these findings, experiments in 6-OHDA-lesioned hemiparkinsonian rats subjected to the priming model have shown that the administration of the D_1_ receptor agonist SKF 38393 on priming expression induces an increase in striatal *zif-268* mRNA (Frau et al., 2013). Moreover, analysis at the single-cell level showed that only enkephalin(−) striatonigral neurons, which belong to the direct pathway, displayed a significantly higher expression of *zif-268* following SKF 38393. On the other hand, enkephalin(+) striatopallidal neurons, which belong to the indirect pathway, that is less involved in abnormal motor responses elicited by dopaminergic drugs (Carta et al., 2010), did not show any significant modifications in the levels of *zif-268*. No significant differences in this effect were observed when the results from primed rats that performed rotational behavior during priming induction were compared with those obtained in rats that were immobilized on priming induction (Frau et al., 2013). This latter finding seems to suggest that modifications in *zif-268* mRNA levels observed in the enkephalin(−) striatonigral neurons of hemiparkinsonian rats during priming expression are not influenced by the fact that the rats could perform rotational behavior during priming induction. In this regard, it is worth mentioning that enkephalin(−) striatopallidal neurons include two subpopulations: substance P(+) and dynorphin(+) neurons. Analysis of *zif-268* in these neuronal populations has demonstrated a selective increase of this IEG in dynorphin(+) striatonigral neurons of rats primed with SKF 38393 that performed rotational behavior during priming induction, compared with primed rats immobilized on priming induction (Figure 2). This finding demonstrates, in the first place, a critical role of drug-stimulated movement performance in the emergence of neurochemical modifications in striatal neurons of dopamine-denervated rats subjected to repetitive administration of dopaminergic drugs, and that the dynorphin(+) neurons are selectively involved in the long-term modifications caused by early motor performance in the priming model (Frau et al., 2013). Moreover, the intensity of rotational behavior on priming expression was found to correlate positively with the levels of *zif-268* mRNA in dynorphin(+) neurons (Frau et al., 2013). This finding is very interesting, as it indicates a relationship between early performance of drug-stimulated movement and appearance of neurochemical adaptations associated with an abnormal motor response to dopaminergic drugs (Frau et al., 2013). With regard to the modifications in the levels of *zif-268* in the priming model, it is also relevant to observe that *zif-268* is rapidly induced in certain forms of learning, or after long-term potentiation (Lanahan and Worley, 1998; O’Donovan et al., 1999; Tischmeyer and Grimm, 1999; Bozon et al., 2002), and has recently been shown to be necessary for the formation of different forms of long-term memory (Jones et al., 2001). Together with the results obtained in the priming model, this finding would provide support to the hypothesis suggesting that abnormal motor responses to repetitive administration of dopaminergic drugs in conditions of dopaminergic denervation might involve the generation of abnormal procedural memories in striatal motor circuits.

## Conclusions

The results obtained in 6-OHDA-lesioned hemiparkinsonian rats subjected to the priming model demonstrate that the early performance of drug-stimulated movement promotes the later emergence of an abnormal motor response and striatal neurochemical adaptations following the subsequent administration of dopaminergic drugs with pro-dyskinetic potential (Simola et al., 2009; Frau et al., 2013). These results may appear to contrast with recent studies in experimental animals and PD patients that demonstrate how performance of movement in the form of physical training and exercise may improve motor deficits and even ameliorate dyskinesias (Goodwin et al., 2008; Döbrössy et al., 2010; Frazzitta et al., 2010; Dutra et al., 2012; Frazzitta et al., 2012; Aguiar et al., 2013). In this regard, it is worth considering that extensive neuroplasticity takes place in the striatum, which regulates movement performance, that physical activity may interfere with these neuroplastic phenomena, eventually influencing the execution of movement at a later time, and that neuroplasticity can be profoundly modified in conditions of dopamine denervation (Tillerson et al., 2001; Packard and Knowlton, 2002; Smith and Zigmond, 2003; Schouenborg, 2004; Graybiel, 2005). Therefore, it can be hypothesized that drug-stimulated movement and voluntary physical activity, given their different nature, might result in distinct neuroplastic adaptations in the dopamine-denervated striatum, leading to different effects on abnormal motor responses. Thus, irrepressible movement stimulated by dopaminergic drugs could overload striatal motor circuits with redundant information and promote pathologic motor learning, eventually triggering the generation of aberrant habits, which may manifest as abnormal motor responses, such as dyskinesias (Calon et al., 2000; Picconi et al., 2005; Jenner, 2008). On the other hand, physical activity in the framework of therapeutic programs could compete with purposeless movements triggered by dopaminergic drugs, therefore counteracting the generation of abnormal procedural mnemonic traces in the striatum, and thus ameliorating motor performance and mitigating dyskinesias.

In summary, the results obtained in hemiparkinsonian rats subjected to the priming model suggest that the performance of movement in response to an initial stimulation of dopamine receptors in the dopamine-denervated striatum plays a key role in the emergence of both abnormal motor responses and specific neuroadaptive changes in response to a later dopaminergic challenge. These results may help understand the initial molecular events that are at the basis of motor complications, such as dyskinesia, associated with DRT used to manage PD.

## Author contributions

Nicola Simola and Lucia Frau: writing of the first draft of the manuscript and review. Giuseppe Frazzitta and Micaela Morelli: manuscript review and critique.

## Conflict of interest statement

The authors declare that the research was conducted in the absence of any commercial or financial relationships that could be construed as a potential conflict of interest.
